# Insight into anomalous hydrogen adsorption on rare earth metal decorated on 2-dimensional hexagonal boron nitride: a density functional theory study†

**DOI:** 10.1039/d0ra01835j

**Published:** 2020-03-31

**Authors:** Shreeja Das, Saroj K. Nayak, Kisor K. Sahu

**Affiliations:** School of Minerals, Metallurgical and Materials Engineering, Indian Institute of Technology Bhubaneswar India kisorsahu@iitbbs.ac.in; Centre of Excellence for Novel Energy Materials, Indian Institute of Technology Bhubaneswar India; School of Basic Sciences, Indian Institute of Technology Bhubaneswar India

## Abstract

Hydrogen interaction with metal atoms is of prime focus for many energy related applications like hydrogen storage, hydrogen evolution using catalysis, *etc.* Although hydrogen binding with many main group alkaline and transition metals is quite well understood, its binding properties with lanthanides are not well reported. In this article, by density functional theory studies, we show how a rare earth metal, cerium, binds with hydrogen when decorated over a heteropolar 2D material, hexagonal boron nitride. Each cerium adatom is found to bind eight hydrogen molecules which is a much higher number than has been reported for transition metal atoms. However, the highest binding energy occurs at four hydrogen molecules. This anomaly, therefore, is investigated in the present article using first-principles calculations. The number density of hydrogen molecules adsorbed over the cerium adatom is explained by investigating the electronic charge volume interactions owing to a unique geometrical arrangement of the guest hydrogen molecules. The importance of geometrical encapsulation in enhancing electronic interactions is explained.

## Abstract 2

The central rare earth cerium atom and underlying apolar B–N bonds in two-dimensional hexagonal boron nitride facilitate a unique arrangement of hydrogen molecules which leads to fairly strong adsorption of eight hydrogen molecules per metal atom.

## Graphical abstract

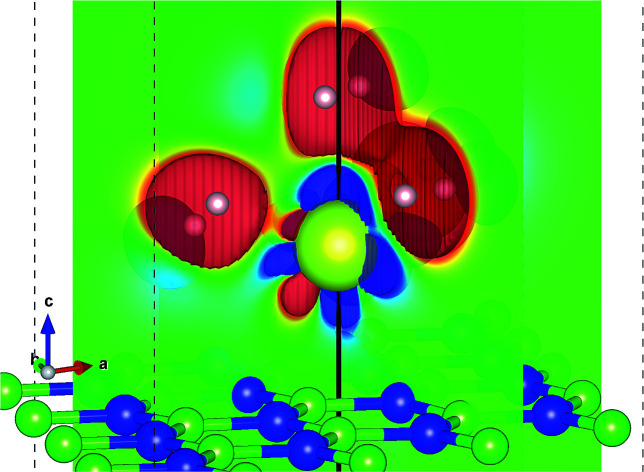

## 1 Introduction

The study of hydrogen interaction with metal atoms has been the core theme for diverse research disciplines, such as finding new materials for hydrogen storage,^1–3^ hydrogen evolution catalysis,^4,5^ corrosion control,^6–8^ chemical synthesis,^9,10^ hydrogen-induced cracking,^11^*etc.* Hydrogen is a ubiquitous element in the universe and the simplest gas but rarely occurs in its pristine gaseous form at ambient conditions. A high-density secondary source of energy, hydrogen also has the potential to replace conventional fossil fuels in the mission to move towards a cleaner energy economy. The study of geometric sites for hydrogen interaction with metal atoms on a two-dimensional (2D) substrate material is one of the most recent and emerging areas for developing materials for energy storage and catalysis.^12^

### 1.1 Interaction of metal and hydrogen

Atomic hydrogen is extremely reactive and readily forms hydrides with most metals. Light metals like Li, Na, and Mg have the advantage of trapping more hydrogen per unit weight of the host material and are, therefore, extremely promising for hydrogen storage materials. Hence, extensive studies on the absorption/adsorption behavior of atomic as well as molecular hydrogen have been performed on these metals.^13–17^ On the other hand, the study of 3d, 4d, and 5d transition metals' interaction with hydrogen is essential to develop low-cost green chemical manufacturing processes.^18^ Another application of studying hydrogen interaction with transition metals is in catalysis and hydrogen evolution from water.^19^ Transition metals are characterized by the presence of many d atomic orbitals for electrons. For early transition metals like Ti, the 18-electron rule, generally applicable for predicting the formation of organometallic compounds, has been successfully used to determine the maximum number of hydrogen molecules adsorbed.^20^Table 1 enumerates the number of hydrogen molecules adsorbed and the hydrogen storage capacities (HSC) on a few metal atoms supported on graphene or an hexagonal boron nitride (h-BN) precursor, borazine, as reported by various studies.

**Table 1:** Comparison of the hydrogen storage capacities (HSC) and number density of hydrogen molecules adsorbed per metal adatom decorated on graphene and borazine

Substrate	Metal adatom	HSC wt%	|Average adsorption energy^a^ (eV per atom)|	No. of H_2_/adatom	Ref.
Graphene	Li	12.8	0.2	4	21
	Ca	8.4	0.3	4	22
	Be, Mg, Fe, Cr, Mo	0	0	0	22
	Pd	3.622	0.879	4	23
	Al	13.79	0.172	6 (maximum *E*_b_ achieved at 4)	24
	Sc	8	0.17	4	25
	Ti	7.8	0.43	4	25 and 26
	V	7.5	0.33	4	25
Borazine (B_3_N_3_H_6_)	Sc	6	0.14	4	27
	Ti	5.9	0.73	4	27
	V	5.8	0.69	4	27

a Absolute value of binding energies reported in the references are recorded here.

Moving further into the periodic table, lanthanides and actinides additionally have unfilled f atomic orbitals for electrons. Very few studies have been performed for studying hydrogen binding properties of these heavy rare earth (RE) metals decorated on 2D materials. Unlike the general 18-electron rule for the main group transition metals, no such indicative rule exists for the lanthanides. It will be interesting to study the effect of their large atomic size, the presence of f-orbitals, and their compounded effect, if any, alongside the d-electrons on the number of hydrogen molecules adsorbed. Density functional theory (DFT) and scanning tunneling microscope (STM) analysis of adsorption of RE metals Eu, Gd, Nd, and Yb on pristine graphene have provided valuable insight into their growth morphology, adsorption mechanism, electronic and magnetic properties without, however, exploring their interaction with hydrogen.^28^ Cerium is the most abundant RE metal with a lot of unfilled d and f-orbitals for electronic interaction. Thus, by taking cerium as the template element over a 2D material substrate, we assess the geometrical aspect of hydrogen interaction with rare earth metals.

### 1.2 Interaction between 2D materials and hydrogen

The choice of substrate for the metal atom decoration is essential for tuning the geometric sites for hydrogen adsorption. 2D materials provide the required expanse of surface area for interaction with hydrogen molecules. Molecular hydrogen in gaseous form can also be adsorbed on material scaffolding to design high-density on-board hydrogen storage materials for mobile vehicle applications. The electron-rich surface provided by graphene and pristine carbon nanostructures was long thought to be ideal for adsorbing large amounts of hydrogen gas for storage. However, later few studies unequivocally agreed that pristine 2D materials and their nanostructures alone cannot serve as practical hydrogen storage materials due to the weak van der Waals forces resulting in very low gravimetric density after adsorption.^29–31^ Intercalation of molecular hydrogen within graphitic layers was also found to be negligible since it is largely localized to the outer exposed surfaces of the bulk material.^32^ It was found by theoretical studies that another 2D material hexagonal boron nitride (h-BN) can bind hydrogen stronger than graphene.^33^ It has been shown that boron nitride nanotubes (BNNTs) are superior (3 wt% HSC) than carbon nanotubes (CNTs) (0.2 wt% HSC) for hydrogen storage.^34–36^ This improved performance is attributed to the heteropolar nature of B–N bonds being able to more strongly bind hydrogen than the apolar C–C bonds in graphene, providing additional exploitable materials design flexibilities.

### 1.3 Interaction of hydrogen with metal adatom decorated 2D materials

Pristine graphene, CNTs, and fullerenes have been experimentally shown to have low hydrogen storage capacity (HSC) ranging from 0.3 wt% to 3.1 wt% only.^2,26,37–39^ This calls for an additional strategy to engineer the geometry of 2D surfaces to help in augmenting the electronic interaction with hydrogen molecules and can be achieved by introducing surface modifications in the form of foreign metal doping or decoration. Other strategies to create geometrical sites for adsorption like vacancies, protrusions, heteroatom doping, edge modifications, wrinkles, interlayer spacing are being continuously explored.^12^ The simple case of decorating metal atoms on high surface area nanomaterials like fullerenes, nanotubes, and 2D sheets show marked improvements in hydrogen binding. Pd, Ru, Ti, Ni all cause considerable improvement in binding energy of hydrogen molecules on the modified carbon-nanostructures.^2,39^ Not limited to carbon, this trend also extends to high surface boron nitride nanostructures as well.^27,40,41^ Recent experimental studies on low cost Mn and V decorated graphene exhibit a storage capacity of 1.81 wt%, a huge improvement over pristine graphene which exhibits 0.25 wt% capacity.^42^ Increased surface area in Au and Pt-decorated graphene have been shown to enhance glucose sensing for application as biosensors and also for gas sensing.^43,44^ Moreover, with respect to hydrogen storage, Ce addition to metallic superalloys have shown good enhancement.^45,46^ Since the bonding characteristics of RE metals is different from that of s and p block simple metals and transition metals, the study of RE adatom adsorption on h-BN sheds additional light on its effect on hydrogen adsorption. This work hence focuses on the usage of RE metal decorated h-BN as a hydrogen storage material and the mechanism behind the hydrogen molecules attached to each decorative atom. Ce decorated boron nitride nanotubes (BNNTs) have been studied using DFT techniques^47^ but the geometrical aspects of the H_2_-metal adatom–substrate interaction are not well understood. Even though lanthanides have higher cohesive energy than transition metals making them more prone to clustering, the present DFT study on their influence over hydrogen storage capacities of h-BN exposes hitherto unfound effects. Additionally, this article probes the underlying reason why binding energy is maximum in the case of four physisorbed hydrogen molecules on Ce-decorated h-BN and goes on to accept much more beyond four hydrogen molecules.

## 2 Results and discussion

### 2.1 Adsorption of Ce on h-BN

**Fig. 1:**
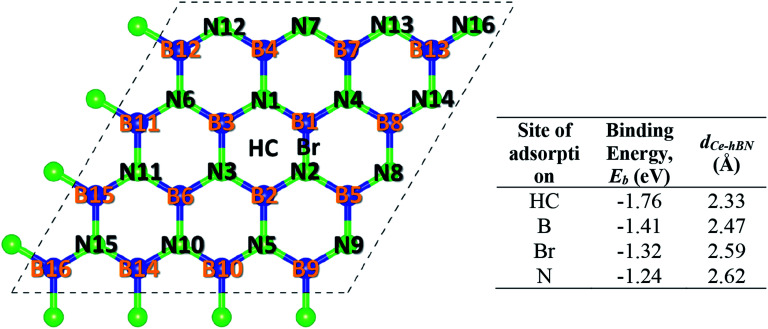
Different adsorption sites for Ce. The system comprises of a 4 × 4 × 1 unit cell of h-BN. Blue (dark) and neon green (light) balls represent boron and nitrogen atoms, respectively. Each atom in a unit cell is identified by a unique index labeled on top of it. The four sites of adsorption: HC (hollow hexagon center), B (on top of any B atom), N (on top of any N atom), and Br (on top of the midpoint of B–N bridge) are also labeled. The corresponding table lists the binding energy (*E*_b_) of Ce at the four different sites on h-BN and the perpendicular distance of the Ce adatom from the basal plane of h-BN (*d*_Ce–hBN_).

Cerium adatom adsorption on h-BN was investigated at four possible sites: on the top of hollow hexagon center (HC), boron atom (B), nitrogen atom (N) and bridge of boron–nitrogen bond (Br), which are depicted in Fig. 1. A measure of the strength of Ce binding on the substrate h-BN is evaluated by eqn (1).1
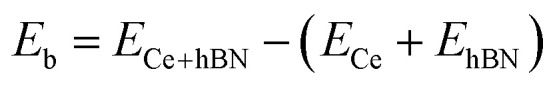
where, *E*_Ce+hBN_, *E*_Ce_, *E*_hBN_, are the total energies of the Ce atom adsorbed on h-BN, a single isolated Ce atom, and the h-BN substrate respectively. Here, more negative values of the binding energy, *E*_b_, indicate stronger binding.

**Fig. 2:**
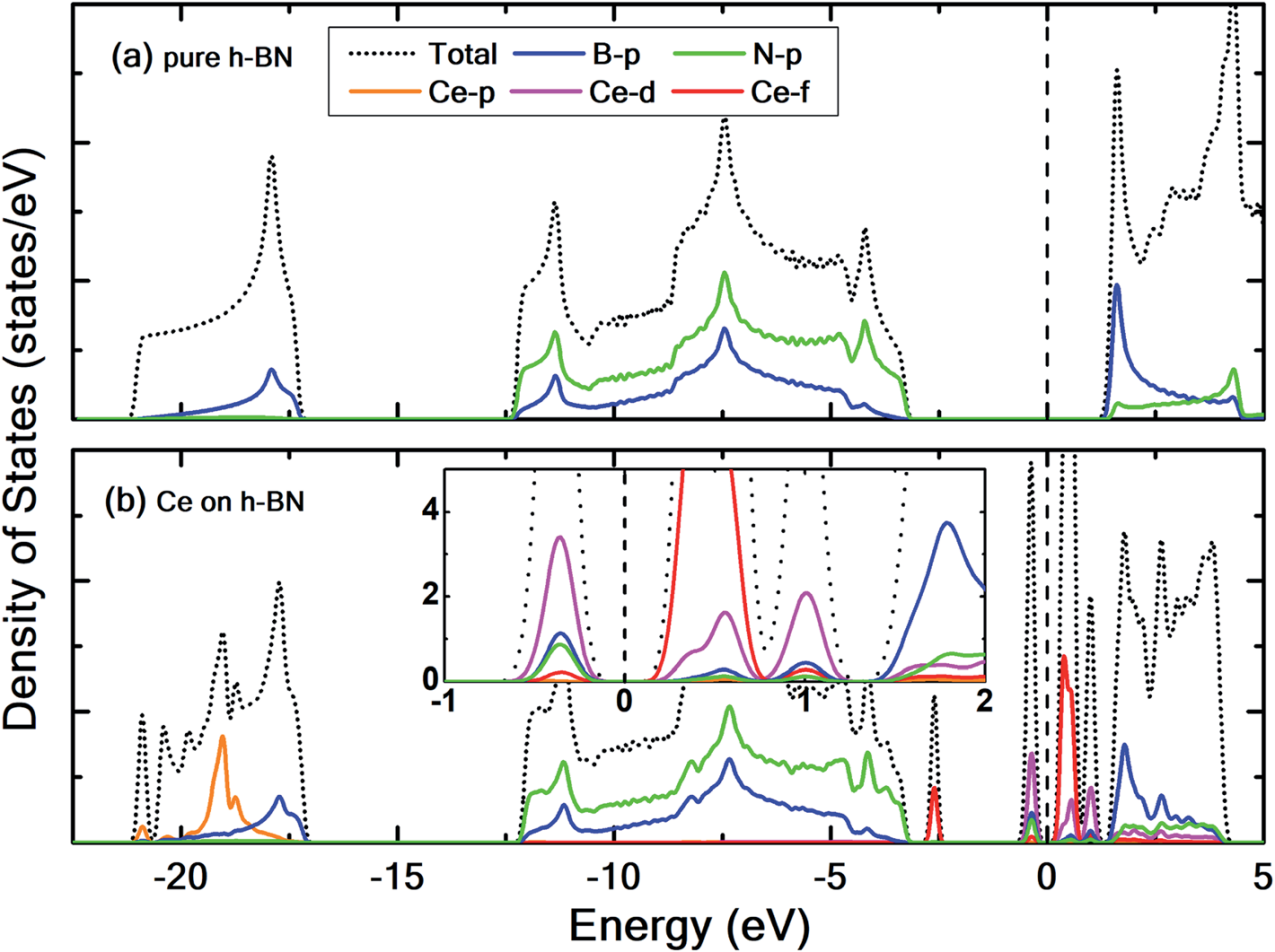
Contributions of Ce p, d, and f orbitals, B-p and N-p orbitals to the total density of states for (a) pristine h-BN and (b) Ce adsorbed on h-BN. Inset in (b) shows only the states near the Fermi level. The dashed vertical line represents the Fermi level which is set to 0 eV.

To prevent the clustering of Ce atoms, a large supercell (4 × 4 × 1) of h-BN is used, wherein the distance between adjacent Ce atoms in the periodic boundary scheme is 10.08 Å. This is large enough to prevent interatomic interactions between the Ce atoms and thus avoid metal cohesion. Fig. 1 shows the four possible sites of adsorption on h-BN taken into account in this study. The values of binding energy of Ce are calculated at the aforementioned sites, out of which Ce is found to bind strongest at the site HC with a binding energy of −1.76 eV. This result is reminiscent of other transition metal atoms like Rh, which also favor the HC site for adsorption on h-BN.^48^ Binding of Ce on h-BN is markedly stronger than most other metal like Au, Pt, Ir, *etc.*^49^ (given in Table S1.1 of ESI†). Such strong binding characteristics can be explained as an effect of enhanced charge transfer between Ce p, d states and the π-orbitals of the adjacent B and N atoms. The partial density of states of Ce adsorbed h-BN with *U* = 5 eV is shown in Fig. 2.

**Fig. 3:**
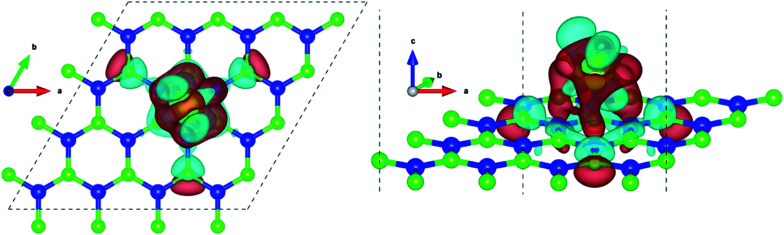
Charge density difference isosurfaces of Ce doped h-BN from different visual angles. Red regions denote charge accumulation and cyan regions denote charge depletion, this, however, has no relation to the color scheme used for different atoms (blue and neon green represent boron and nitrogen respectively). Isosurface level = 0.0045 electrons per bohr^3^.

Pristine h-BN is a wide-gap insulator with a calculated bandgap of at least 4.5 eV, which is in agreement with other theoretical studies.^50,51^ The addition of Ce introduces extra occupied and unoccupied states near the Fermi level corresponding to the numerous Ce d and f orbitals drastically reducing the bandgap to 0.08 eV. These states are localized around the Fermi level at −2.6 eV (filled Ce-f), −0.4 eV (filled Ce-d) and 0.6 eV (unfilled Ce-d and Ce-f). Notice that pristine h-BN's B–N sp^2^ hybridization is preserved (overlap of B-p and N-p states from −12 eV to −3 eV in Fig. 2(b); B-s states are not shown) even after Ce addition. Electronic interaction and charge transfer behavior are measured by using charge density difference diagrams (Fig. 3) and Bader charge analysis.^52^ Since B is less electronegative than N, it ends up transferring its solo valence electron (in 2p) easily to N as part of the ionic B–N bond. Hence, the B atoms are electron-deficient and have a tendency to attract electrons from any external source, Ce in this case. The interaction between filled π orbitals of h-BN and d/f orbitals of Ce appears at about −0.4 eV in the inset of Fig. 2. Ultimately, B atoms claim a higher share of electrons from Ce and become less positively charged than in pristine h-BN. This is also corroborated by charge density difference isosurfaces in Fig. 3 depicting regions of electron abundance (red) spanning from the Ce adatom to the adjacent B atoms indicating loss of electrons from Ce to B atoms.

The addition of a heavy atom causes some structural changes in the h-BN substrate. Fig. S4.1† shows the extent of planar warping when Ce is adsorbed at the center (HC). The changes in the position of atoms are an effect of two phenomena – structural strain due to the decoration of a heavy atom and electronic volume interactions. To understand this, both structural changes and charge transfer behavior is studied. A detailed account of this analysis is provided in Sections S4 and S5 of ESI.†

### 2.2 Hydrogen adsorption on Ce/h-BN

**Fig. 4:**
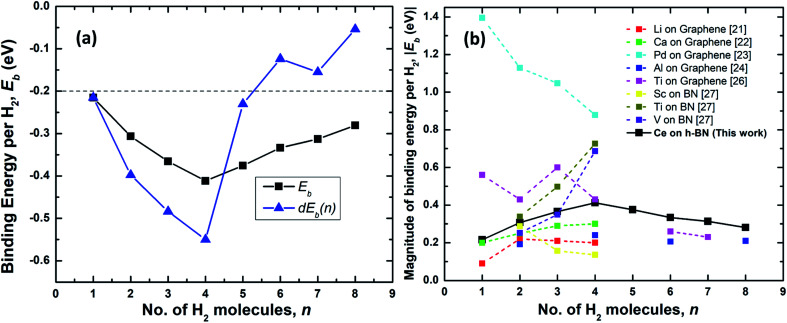
(a) Binding energy per hydrogen molecule on Ce decorated h-BN, *E*_b_^*n*^ (black squares) and energy required to add *n*th hydrogen molecule, d*E*_b_(*n*) (blue triangles) over Ce decorated h-BN. The dashed line represents the upper limit of the US DOE target for ambient hydrogen storage; the lower limit being at −0.7 eV.^53^ (b) Comparison of the absolute value of average binding energy, |*E*_b_| as number of hydrogen molecules is increased for other metals decorated on 2D materials – graphene and borazine as reported in literature.

Hydrogen physisorption on Ce-decorated h-BN was then studied by calculating the binding energy per hydrogen molecule as the number of hydrogen molecules is increased from *n* = 1 to 8. The binding energy per hydrogen molecule is given by eqn (2).2
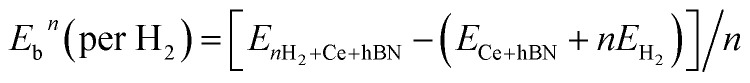
where *E*_*n*H_2_+Ce+hBN_, *E*_Ce+hBN_, *E*_H_2__, are the ground state total energies of *n* hydrogen molecules adsorbed on Ce-decorated h-BN, only Ce-decorated h-BN, and a single isolated hydrogen molecule respectively. The additional energy that binds each subsequent hydrogen molecule, denoted by d*E*_b_(*n*) is calculated using eqn (3).3
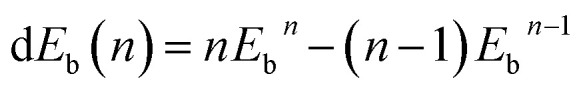
Both *E*_b_^*n*^ and d*E*_b_(*n*) as a function of *n* is shown in Fig. 4(a).

The physisorption binding energies of hydrogen on various metals (alkaline and transition) decorated on 2D materials are compared with the binding on Ce in Fig. 4(b). Most of the metal atoms are seen to be able to bind up to 4 hydrogen molecules. Ti and Al exhibit weaker binding with 6H_2_ molecules *i.e.* 0.26 eV and 0.21 eV respectively compared to 0.33 eV for Ce.^24,26^ It is possibly because these previous studies have not considered dispersion corrections. However, it is worthwhile to note that Liu *et al.*^26^ report that a fifth H_2_ molecule does not bind at all with the Ti atom and escapes away. Also, in other studies, Ti adsorbed on a six-membered ring of C60 has been shown to obey the 18-electron rule and adsorb only 4 hydrogen molecules.^20,39^ The binding energies reported for Al by Ao and Peeters^24^ are for the case when both sides of graphene are decorated with two Al atoms.

Hydrogen adsorption on both Ti and V decorated BN-based borazine shows a trend of increasing binding energies as *n* is increased from 2 to 4 (the first hydrogen molecule dissociates to form a dihydride complex). Cr, Mn and Fe causes dissociation of the hydrogen interatomic bond to form dihydrides *via* chemisorption and does not facilitate physisorption at all.^27^ Thus, in comparison, a lone Ce atom is able to easily adsorb 4 hydrogen molecules without dissociation and with increasing binding energies. Remarkably, subsequently added 5th to 8th molecules are also quite strongly bound making it an interesting case for further studies on the hydrogen adsorption properties of lanthanide elements.

As shown in Fig. 4(a), the binding energies all lie well within the recommended range of −0.2 eV to −0.7 eV for ambient hydrogen adsorption and desorption.^53^Table 1 records the corresponding adsorption energies of hydrogen molecules on various metal decorated 2D materials. The magnitude of binding energies of hydrogen reported on alkaline metals like Li and Ca and transition metals like Sc, Ti and V mostly lie within the range of 0.16 eV to 0.70 eV, with the exception of Pd which has the highest binding energy of about 0.88 eV. Thus, in comparison, Ce with a highest binding energy magnitude of 0.41 eV at *n* = 4, is reasonably good at binding molecular hydrogen. Additionally, each Ce adatom in a 4 × 4 × 1 supercell of h-BN is able to attract at least eight hydrogen molecules favorably. Binding energy is highest when *n* = 4. Interestingly, four also seems to be the magic number of hydrogen molecules physisorbed on many other transition metal adatoms over the h-BN precursor, borazine or graphene, like Sc, Ti, V, Pd, Al, as reported by various other theoretical studies (Table 1). Most transition metal atoms follow the 18-electron rule (or effective atomic number rule) to estimate the number of hydrogen molecules adsorbed.^20^ However, since such a rule is not expected to work for 4f elements like cerium, it is surprising that the binding is highest for Ce also at 4 hydrogen molecules as predicted by the 18-electron rule. This is because 4f shells of lanthanides are too compact and shielded^54^ to allow active interaction with hydrogen molecules and the d electrons are mostly involved in binding with the hydrogen molecules. However, unlike main group transition elements, the number of hydrogen molecules is not limited to four but increases up to eight, albeit with progressively decreasing binding energies. A detailed explanation of the reason behind the increased number of hydrogen molecules beyond the number four is thus provided below.

**Fig. 5:**
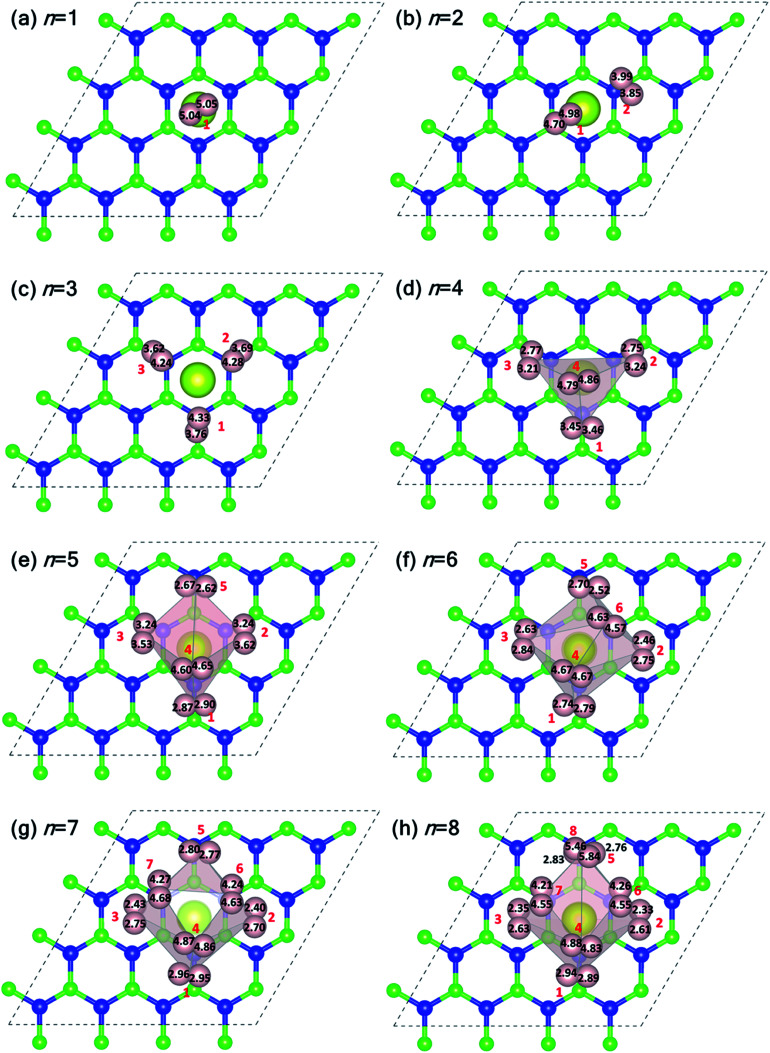
(a–h) Equilibrium structures of hydrogen molecules physisorbed on Ce decorated h-BN in a plan view scheme as the number of hydrogen molecules is increased from *n* = 1 to 8. The peach-colored balls represent hydrogen atoms with an enhanced radius of 1.2 Å for better visibility. Light pink surfaces are Delaunay tetrahedra with the center of gravity of the hydrogen molecules as vertices. Integers (in red) denote the index of the hydrogen molecule, *n*. The decimal numbers in black color on each hydrogen atom denotes their *z*-elevation.

Fig. 5 shows the plan-views of the stable configurations of hydrogen molecules (peach-colored balls; radius increased to 1.2 Å for better visibility) adsorbed on Ce decorated h-BN as *n* is increased from 1 to 8. The integers in red denote the index of each molecule for easy identification. The decimal numbers printed on each hydrogen atom indicate its normal distance from the basal plane of h-BN (*z*-distance). The first hydrogen molecule lies horizontally at about 5 Å from the basal plane right on top of the Ce adatom (Fig. 5(a)). It was seen to prefer a horizontal alignment over vertical alignment with respect to the basal plane, indicating that the H–H σ electrons are more exposed to Ce for bonding in this alignment. When one more hydrogen molecule is added, it positions itself on top of the heteropolar B–N bond and pushes the first hydrogen molecule away from HC and slightly downwards as well (Fig. 5(b)). An interesting event happens when one further hydrogen molecule is added (Fig. 5(c)). Now, all the 3 hydrogen molecules are seen to align specifically on the B–N bonds involving only the nearest B atoms from HC. These particular nearest B–N bonds thus become anchoring points for hydrogen molecule adsorption and this is discussed in detail in the following. The addition of a 4th molecule (Fig. 5(d)), further increases the binding strength of each molecule due to an interesting phenomenon where the previous three hydrogen molecules are pushed downwards (decrease in *z*-distance), and the 4th molecule settles on top of the Ce adatom. The tendency to bind is so strong that, the three anchoring hydrogen molecules are actually twisted from their previous stable positions parallel to the B–N bond and now lie across the B–N bonds. Thus, all four hydrogen molecules form a tetrahedral tent-like structure that encompasses the central Ce adatom. The average *z*-elevation of the 4th hydrogen molecule in this case (Fig. 5(d)) is 4.83 Å, which is much lower than the case for a single hydrogen molecule, which is 5.05 Å. This is the most optimal structure, and the clue behind the reason for this can be obtained from the 18-electron rule as well as Fig. 3, which suggests a correspondence between the locations of hydrogen molecules with that of the charge transfer lobes between Ce and h-BN. Hydrogen molecules gain some excess negative charge from the Ce adatom. The regions of excess charge in Fig. 3 (red lobes) get involved in bonding with the electron-rich hydrogen molecules giving rise to a stable arrangement of hydrogen molecules encompassing the central metal atom.

Introducing the 5th molecule causes the average binding energy to suddenly decrease (black squares in Fig. 4(a)). The individual binding energy of the 5th molecule is drastically reduced (blue triangles in Fig. 4(a)), indicating it is an unwelcome addition to the stable 4H_2_ + Ce/h-BN configuration. Addition of subsequent 6th, 7th, and 8th hydrogen molecules further witnesses a decrease in both average binding energy and individual binding energies (Fig. 4(a)). Structurally, they start forming a two-tiered cage-like structure around the Ce atom. The hydrogen molecules labeled *n* = 1, 2, 3, 5 constitute the lower tier, and *n* = 4, 6, 7, 8 constitute the upper tier (Fig. 5(h)). Note that the lower tier of hydrogen molecules has been pushed down towards the basal h-BN plane by the upper tier of hydrogen molecules due to steric hindrance. A visual representation of the charge landscape in the form of charge density difference diagrams is presented in Fig. S6.1 of ESI.†

**Fig. 6:**
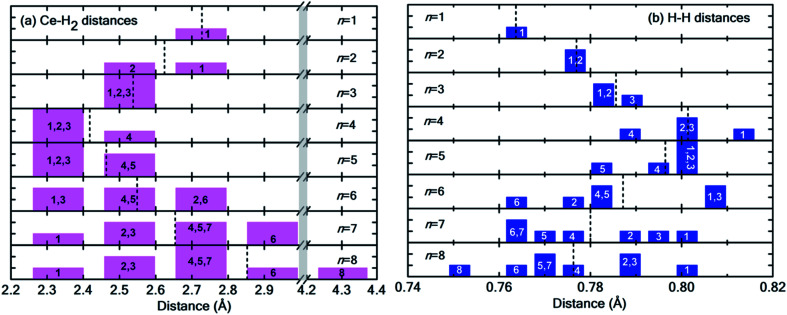
(a) Distribution of Ce–H_2_ distances for *n* = 1 to 8H_2_ molecules. Notice the break in the plot from 3.0 Å to 4.2 Å indicated by the vertical grey strip. (b) Distribution of H–H interatomic distance in H_2_ molecules. In both histograms, the integer index of hydrogen molecules is printed in each bar, and the dotted black lines represent the average values. Please note that both Fig. 5 and this figure use identical indexing for H_2_ molecules.

Fig. 6(a) and (b) show the distribution of Ce–H_2_ and H–H distances, respectively. It is worthwhile to note that, in general, with decreasing Ce–H_2_ distances, the H–H interatomic distance increases and *vice versa*. Until *n* = 4, the average Ce–H_2_ distance decreases with hydrogen molecules enclosing upon Ce atom, bringing about an elongation in average H–H bond length (Fig. 6(b)). Such bond elongation in hydrogen molecules is proof of stronger binding with the Ce adatom. Beyond *n* = 4, the process is reversed, indicating weakening binding, as reflected in Fig. 4(a). This peculiar behavior is possibly due to Kubas type interaction between Ce–H_2_ coupled with H_2_–H_2_ van der Waals bonding dominating until *n* = 4, after which dispersion forces take precedence because of the unique geometrical ordering as discussed earlier causing hydrogen molecules to drift away from Ce. The 8th molecule is seemingly unaffected (in terms of bond elongation) by the Ce adatom since it is quite far away from it. It is conjectured that the combined effect of unfilled d orbitals, substrate π orbitals and the hydrogen–hydrogen intermolecular interaction in a unique geometrical arrangement, assisted by the large size of the adatom, can explain such systems' affinity for a count of hydrogen molecules much more than four.

**Fig. 7:**
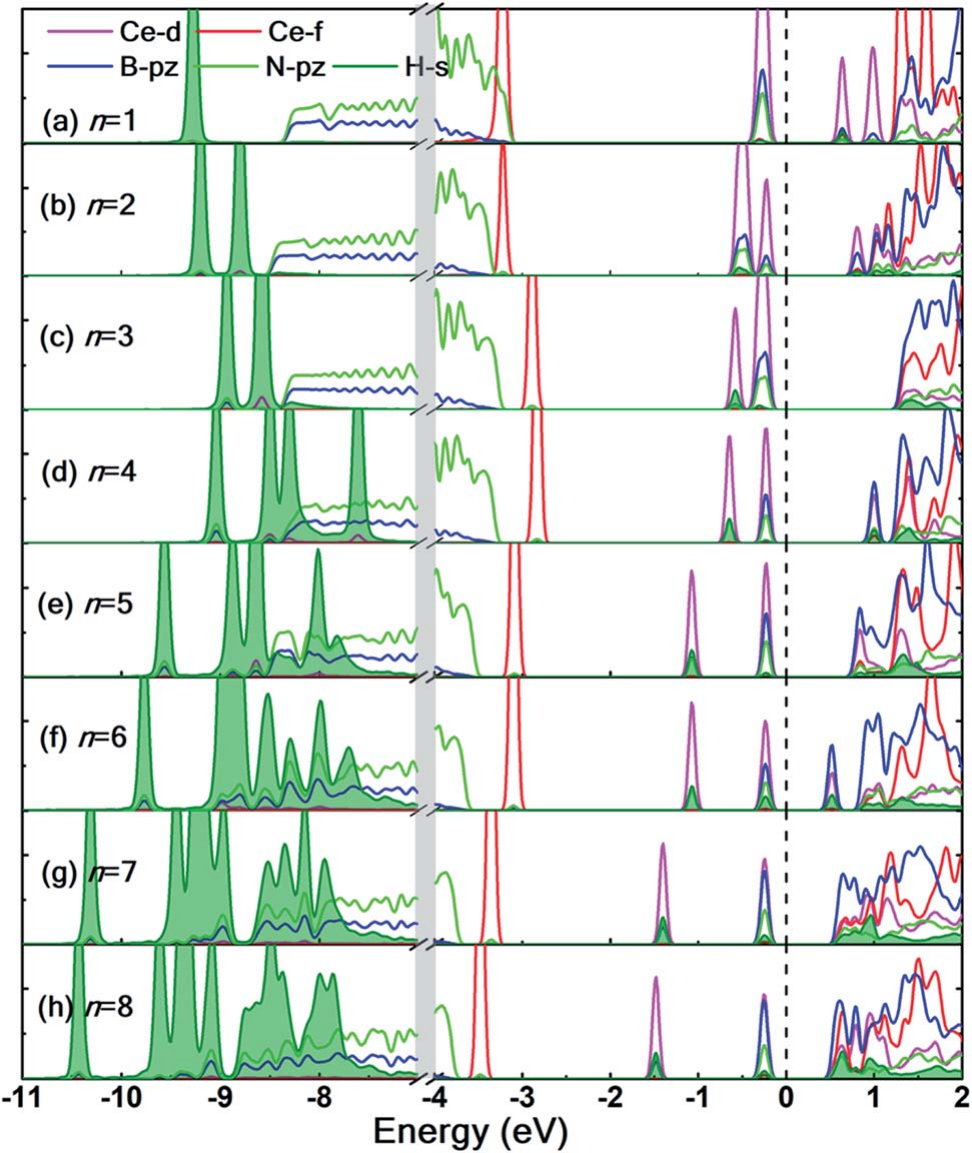
(a–h) Partial density of states for *n* = 1 to 8 hydrogen molecules adsorbed on Ce decorated h-BN. Notice the break in plot from −7 eV to −4 eV indicated by grey vertical strip. The contributions of H-s orbitals to the total density of states are highlighted by the filled area (in green) under the curve. Fermi level is set at 0 eV.

The partial density of states (PDOS) of the hydrogen adsorbed Ce decorated h-BN is shown in Fig. 7 to further understand the electronic interaction of hydrogen molecules and Ce adsorbed h-BN. The focus is specifically on the Ce-d and f, BN-π (B-pz, N-pz), and H_2_-σ (H-s) orbitals. The charge interactions can be divided into three main categories: (i) Ce-d and H_2_-σ, (ii) BN-π and H_2_-σ, and (iii) H_2_-σ and H_2_-σ. In general, below the Fermi level, H_2_-σ electrons strongly interact with Ce-d and BN-π electrons as indicated by the localized peaks near the Fermi level. As the number of hydrogen molecules is increased, the filled Ce-d levels split and start bonding with H_2_ bonding σ orbitals, which causes those Ce-d levels to shift downwards in energy towards the core levels (peaks shifting from −0.5 eV to −2 eV in Fig. 7(a–h)). For *n* = 1, the H_2_-σ levels are seen to not interact too much with Ce-d or BN-π orbitals. It is almost as if the one molecule is very slightly affected by the Ce adatom. This also gets reflected in considerably low binding energy (Fig. 4(a)) and very minor bond elongation (Fig. 6(b)). It is only when the number of hydrogen molecules is increased (noticeably after *n* = 3), that there is a notable charge transfer between the Ce-d and the adsorbed hydrogen molecules (−0.5 eV to −1.5 eV in Fig. 7(d–h)). The charge transfer between H_2_-σ orbitals and Ce-d levels is reminiscent of typical Kubas interactions. In such type of interactions, transition metal atoms' d-orbital donate electronic charge to H_2_-σ orbitals and, in return, H_2_-σ* anti-bonding orbitals back-donate electrons to the metal-d levels. Similarly, from PDOS analysis, we can hypothesize that the Ce atom and hydrogen molecules bind *via* Kubas type interaction albeit with significant assistance from the underlying BN-π orbitals (presence of congruent Ce-d, B-pz, N-pz and H-s peaks from 0 eV to −2 eV in Fig. 7), all leading to stronger hydrogen binding.

H_2_-σ bonding orbitals are marked by the olive-green peaks deep into the valence band (from −7 eV to −11 eV). While for *n* = 1, 2, 3, these peaks are markedly separated from each other. At *n* = 4, these peaks start merging, also termed as band broadening, which is an indicator of heightened H_2_–H_2_ intermolecular charge transfer. The visual evidence is also present in the charge density difference diagrams (Fig. S6.1(c and d)†) where the regions of electron abundance are disjointed until *n* = 3. This intermolecular interaction again augments the binding energy of hydrogen molecules to the substrate (for *n* = 4 only) until steric hindrance due to geometrical constraints acts as a counteraction (*n* > 4).

**Fig. 8:**
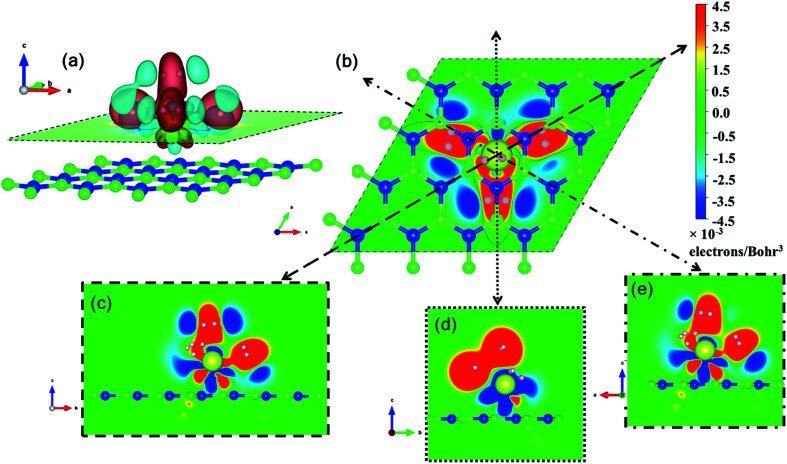
Three and two dimensional charge density difference for *n* = 4 hydrogen molecules. (a) 3D charge density difference isosurfaces with red regions indicating charge accumulation and cyan regions indicating charge depletion. (b) 2D projection of the charge density difference on the plane indicated by the dashed line in (a). (c–e) Projection on various planes indicated by the dotted lines in (b).

Ce, like many other transition metal atoms, also adsorbs exactly four hydrogen molecules with the highest binding energy. Fig. 8 gives a closer look at the charge density difference isosurfaces of specifically the 4 hydrogen molecules adsorbed Ce decorated h-BN (*i.e. n* = 4). The H_2_-σ electron volume is seen to align along the three anchoring points over the nearest B–N bonds (Fig. 8(b)). Donation of Ce-d electrons to H_2_-σ is apparent in Fig. 8(c and e) with overlapping regions of electron accumulation. The beginnings of H_2_–H_2_ van der Waals interaction is also evident from Fig. 8(d) with joint regions of negative charge abundance (red lobes).

**Fig. 9:**
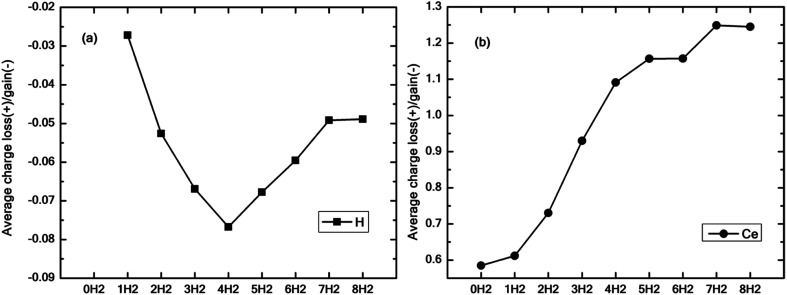
(a) Average electronic charge lost (positive values) and gained (negative values) by all hydrogen atoms for *n* = 1 to 8 on cerium decorated h-BN given by Bader charge analysis. (b) Charge lost by the central Ce adatom in the process of adsorbing 1 to 8 hydrogen molecules.

Fig. 9(a and b) show the average excess charge gained by the hydrogen molecules and Ce adatom as the number of hydrogen molecules is increased from 1 to 8. Fig. 9(a) follows a trend similar to the graph of binding energy (black squares), as shown in Fig. 4(a). For the case of just one molecule, the charge gained by hydrogen is minimal. The average charge gained by the hydrogen molecules increases until *n* = 4, which gives the best binding energy and then starts decreasing thereon. On the contrary, in Fig. 9(b), as the number of molecules is increased, the Ce adatom loses more and more charge to the surrounding hydrogen molecules indicating the presence of charge interaction beyond the stipulated four hydrogen molecules.

Thus, hydrogen adsorption on Ce decorated h-BN is governed not only by the electronic charge of the central rare earth element but to a large extent by the geometry of the cage-like enclosure of hydrogen molecules, which facilitates hydrogen–hydrogen intermolecular interactions. Binding energy increases only until *n* = 4 and then starts decreasing (Fig. 4(a)). Ce being an element with a high atomic radius offers more spatial volume for electronic interaction. This creates opportunities for geometry-driven adsorption of hydrogen molecules, which are able to accommodate favorably around the Ce adatom assisted by the heterogeneous charge landscape due to adjacent B–N bonds. The unique geometrical arrangement due to H_2_–H_2_ interactions causes Ce to continue to transfer charge to an increasing number of hydrogen molecules. Thus, it can be said that even though charge transfer plays a significant role in enhancing the binding of hydrogen molecules, it is their geometrical arrangement that dominates the number density of hydrogen molecules adsorbed around the metal adatom. Ce being the first of rare-earth metals, this observation might be generalized to more such similar systems consisting of later lanthanides.

## 3 Conclusions

In summary, we performed DFT based first-principles calculations on cerium decorated 2D material, h-BN and studied its hydrogen binding capacities. Cerium was found to be strongly bonded with h-BN and helped in significantly enhancing the molecular hydrogen binding energy. We have studied the binding of up to 8 hydrogen molecules on the novel material by incorporating dispersion, dipole, and Hubbard corrections and found that the binding energy per molecule lies within the desired range for ambient adsorption and desorption. Although the lanthanide f-orbitals do not play a major part in increasing binding with hydrogen, the rare earth d-orbitals contribute significantly to improve the binding energy of hydrogen molecules in agreement with the Kubas interaction mechanism.

Furthermore, many other previous studies showed that hydrogen adsorption on transition metal decorated 2D materials maxes at four hydrogen molecules for each adatom. In this study, cerium also showed the strongest binding with exactly four hydrogen molecules. But even after increasing the number of hydrogen molecules to eight, the structure continued to remain stable. This phenomenon was thoroughly investigated, and our analysis suggests that the geometric alignment of hydrogen molecules coupled with hydrogen intermolecular interactions plays a major role in addition to the orbital interactions due to empty d states and heteropolar B–N bonds. This, in turn, not only helps in increasing the binding strength but also increases the number density of hydrogen molecules adsorbed.

## 4 Methods

First-principles calculations for structural optimization, force, and energy calculations were performed using spin-polarized DFT as implemented in the Vienna *ab initio* simulation package (VASP),^55^ which uses a plane wave basis set^56^ and, the projected augmented wave (PAW) method^57^ to account for electron–ion interactions. The generalized gradient approximation (GGA) method by Perdew, Burke, and Ernzerhof (PBE)^58^ is used to treat the electron exchange-correlation interactions because it is much better than LDA^59^ in reproducing long-range dispersion forces like van der Waals interaction. Cut-off energy for the plane-wave basis set is fixed at 650 eV and the convergence in energy at 10^−5^ eV. All atoms are relaxed until the forces on each atom are less than 0.01 eV Å^−1^. The structural optimization was performed with energy and force tolerances of 10^−5^ eV and 0.01 eV Å^−1^ respectively. The optimization was performed in two steps: (a) Ce adatom adsorption on h-BN was studied by allowing all atoms to fully relax without constraints. (b) Once the basal adsorbent structure was fully optimized, their atomic positions were fixed and hydrogen adsorption on Ce decorated h-BN was studied by only allowing the hydrogen molecules to structurally relax until forces on each atom is less than 0.01 eV Å^−1^. The effect of van der Waals (vdW) interactions is included by implementing the empirical correction scheme DFT + D2 as proposed by Grimme.^60^ Dipole corrections in the *z*-direction are also taken into account. The density of states were plotted using Gaussian smearing with a width of 0.05 eV.

For strongly correlated systems, an on-site Coulomb interaction known as Hubbard interaction has to be applied. Accordingly, the Ce f-electrons are localized by applying a Hubbard parameter, U, as given by Dudarev^61^ for the exchange-correlation functional. This on-site interaction is applied only to the f-orbital of Ce. In accordance with other extensive studies on appropriate U values of cerium based compounds,^62,63^ we henceforth adopt a *U* = 5 eV and *J* = 0 eV throughout for subsequent calculations. A comparison of the effect of *U* on the density of states is presented in the ESI Fig. S3.1.†

A 4 × 4 × 1 supercell (32 atoms) of h-BN was constructed, and numerous iterations of electronic and ionic relaxations were performed to arrive at an optimum lattice constant. The lattice parameters of h-BN using the GGA functional were determined to be *a* = *b* = 2.52 Å. The in-plane parameters are very close to the experimental value of 2.51 Å.^64^ A vacuum of 20 Å is taken in the *z*-direction to avoid interlayer interaction due to periodic images. The energy was found to be well converged with a *k*-points mesh size of 9 × 9 × 1 and taken as such for all systems henceforth. Ce pseudopotential with 12 valence electrons: 5s^2^ 5p^6^ 4f^1^ 5d^1^ 6s^2^ was used for this study. All crystal structures and charge density visualization in figures are drawn using the VESTA software.^65^

## Author contributions

The project was planned by K. K. S. and S. K. N. The simulations were carried out by S. D. The manuscript was prepared by S. D., K. K. S., and S. K. N.

## Data and materials availability

All data needed to evaluate the conclusions in the paper are present in the paper and/or the ESI.† Additional data related to this paper may be requested from the authors.

## Conflicts of interest

The authors declare that they have no competing interests.

## Supplementary Material

RA-010-D0RA01835J-s001
