# Supplementary material for: Insight into anomalous hydrogen adsorption on rare earth metal decorated on 2-dimensional hexagonal boron nitride: a density functional theory study
Source: RSC Adv. 2020 Mar 31;10(22):12929–40. doi: 10.1039/d0ra01835j (PMC9051411; doi:10.1039/d0ra01835j)
Supplement: RA-010-D0RA01835J-s001 [file RA-010-D0RA01835J-s001.pdf]

Electronic Supplementary Material (ESI) for RSC Advances.
This journal is © The Royal Society of Chemistry 2020

Supplementary Materials

Insight into anomalous hydrogen adsorption on rare earth metal decorated on 2-dimensional hexagonal Boron Nitride: A density functional theory study

Shreeja Das[1,2], Saroj K. Nayak [2,3], Kisor K. Sahu[1,2*]

[1]School of Minerals, Metallurgical and Materials Engineering, Indian Institute of Technology Bhubaneswar, India.

[2]Centre of Excellence for Novel Energy Materials, Indian Institute of Technology Bhubaneswar, India.

[3]School of Basic Sciences, Indian Institute of Technology Bhubaneswar, India.

*Corresponding author. Email: kisorsahu@iitbbs.ac.in

S1. Binding of other metals on h-BN.

Some studies have been performed on the binding of different metal atoms, mostly transition metals on h-BN. The binding energies of these adatoms is given in Table S1.1

Table S1.1. Binding energies of different metals on h-BN. The reported binding energies of few metal atoms on h-BN[1]. More negative values indicate stronger binding.

Metal adatom	Binding energy (eV) [1][2]
Cu	-0.14
Ag	0.08
Au	-0.03
Pt	-1.51
Rh	-1.03
Pd	-0.94
Fe	-0.52
Co	-0.50

Ir	-1.23

S2. Validation against existing work.

For additional validation, the binding of rare earth element Eu on graphene was computed and compared with previous results[3]. Valence configuration for Eu was taken as $5p^6$ $6s^2$. The binding energies of Eu and Yb on graphene was found to be -0.85 eV and -0.27 eV which is close to reported value of -0.90 eV and -0.32 eV. The slight discrepancy may be due to the difference in the exact nature of pseudopotentials used.

S3. Selection of Hubbard parameter, U.

The effect of U level on the nature of interaction between the adatom and the planar substrate can be found from the partial density of states of the constituent atoms as shown in Fig. S3.1. Plain GGA (with U=0 eV) calculations are known to underestimate the band gap. This is more pronounced for species consisting of highly localized f-electrons like transition metals including lanthanides. Thus, for heavy elements like cerium with highly correlated f electrons, the strong self-interaction terms due to localized d and f electrons are not sufficiently cancelled while applying plain LDA or GGA [4]. To assess the localized nature of these f-electrons, values of the Hubbard parameter, U on the Ce f-electrons are increased through 2, 3 and 5 eV. Without any Hubbard correction, the strongly correlated Ce f-electrons are severely delocalized and indicate spuriously narrower band gaps. Table S3.2 shows the varying band gaps as U levels are increased. As U is increased, new states corresponding to cerium f-orbitals start appearing. Consequently, a band gap of 0.8 eV is observed for Ce adsorbed on h-BN with U correction of 5 eV as compared to 0.22 eV without Hubbard. Indeed, the appropriate value of U is arrived at by empirical comparison with any particular observable like lattice parameter or formation energy or band gap or bulk modulus etc. [2], [5]. Thus, the choice of U is widely debated and is dictated mainly by the measured property. Without delving into an in-depth study, in accordance with other extensive studies on appropriate U values of Cerium based compounds [6], [7] we henceforth adopt a U = 5 eV and J = 0 eV throughout for all calculations.

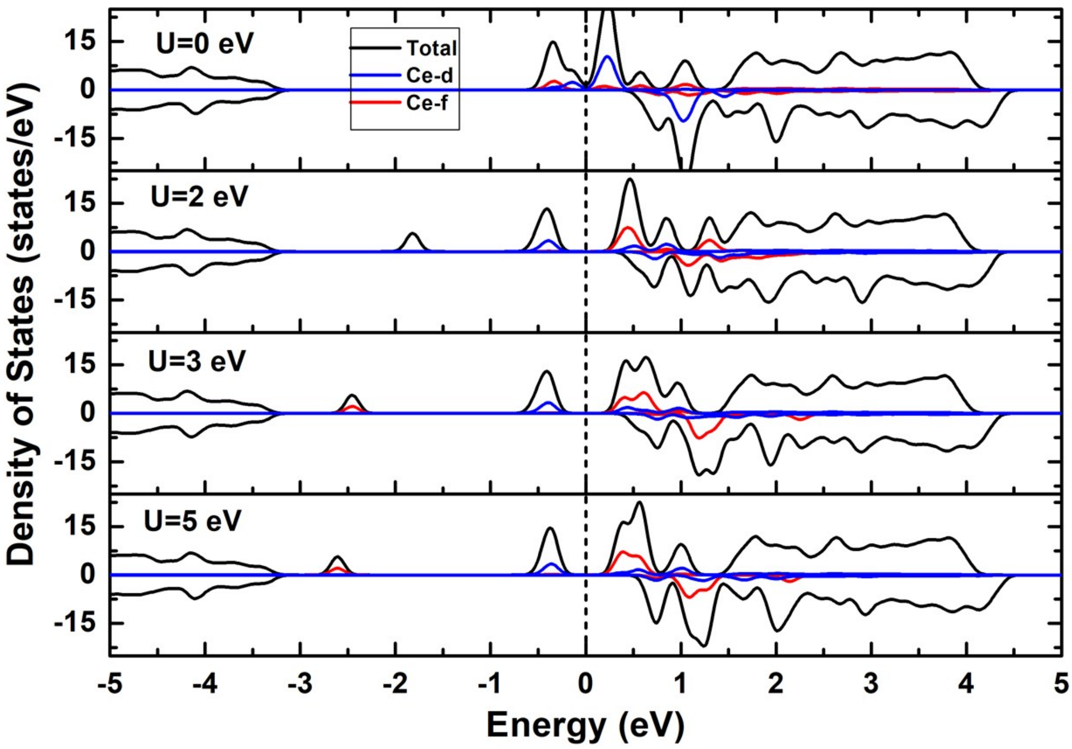

Fig. S3.1. Partial density of states at different levels of U. This figure shows the partial density of states contribution of Ce d and f electrons as the Hubbard correction parameter, U is increased through 0, 2, 3 and 5 eV.

Table S3.2: Band gaps of Ce adsorbed h-BN at different U levels. The band gaps observed for Ce adsorbed h-BN as a function of U parameter.

**U (eV)**	**Band Gap (eV)**
0	0.18
2	0.35
3	0.79
5	0.79

S4. Ce on h-BN optimum structure (HC).

The optimum coordinates for cerium adsorbed on h-BN is provided in the VASP input file format (POSCAR):

```
h-BN 4x4 with Ce at HC
1.0
+8.7264000000  -5.0378800000  +0.0000000000
+8.7264000000  5.0378800000  +0.0000000000
+0.0000000000  +0.0000000000 +20.0000000000
B N Ce
16 16 1
Cartesian
+1.4543599988  +0.0000000000  +5.0221725871
+3.6360206300  +1.2595078582  +5.0221665136
+5.8151941528  +2.5236277153  +4.9824915824
+7.9963500672  +3.7828461417  +4.9824998022
+3.6360206300  -1.2595078582  +5.0221665136
+5.8101712087  +0.0000000000  +5.0250396154
+7.9875211235  +1.2797009346  +4.9882971209
+10.1845114026  +2.5253781472  +5.0250394957
+5.8151941528  -2.5236277153  +4.9824915824
+7.9875211235  -1.2797009346  +4.9882971209
+10.2041691033  +0.0000000000  +4.9882747873
+12.3676819248  +1.2592199281  +4.9824854709
+7.9963500672  -3.7828461417  +4.9824998022
+10.1845114026  -2.5253781472  +5.0250394957
+12.3676819248  -1.2592199281  +4.9824854709
+14.5440073343  +0.0000000000  +4.9771027324
+2.9087824075  +0.0000000000  +5.0348474143
+5.0900423580  +1.2603751802  +5.0130090630
+7.2633783397  +2.5338276542  +4.9596159166
+9.4529732527  +3.7791498883  +5.0130250295
+5.0900423580  -1.2603751802  +5.0130090630
+7.2719275866  +0.0000000000  +4.9890699556
+9.4536083912  +1.2595062463  +4.9890099207
+11.6361439201  +2.5187781005  +5.0130223048
+7.2633783397  -2.5338276542  +4.9596159166
+9.4536083912  -1.2595062463  +4.9890099207
+11.6523947347  +0.0000000000  +4.9596062818
+13.8173693820  +1.2584510409  +4.9804333057
+9.4529732527  -3.7791498883  +5.0130250295
+11.6361439201  -2.5187781005  +5.0130223048
+13.8173693820  -1.2584510409  +4.9804333057
+15.9972083973  +0.0000000000  +4.9804473453
+8.7265893331  +0.0000000000  +7.3212482287
```

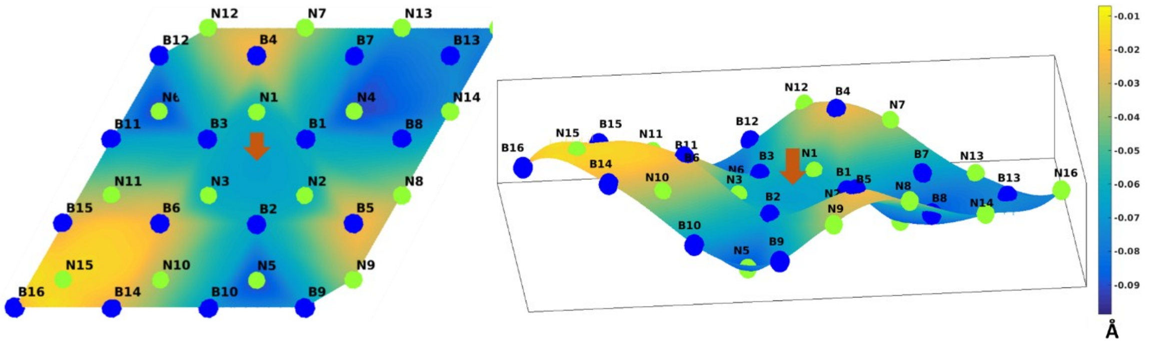

Fig. S4.1. Planar warping of h-BN. Color-mapped image of the z-axis displacement of the h-BN substrate. Blue (dark) and green (light) circles denote boron and nitrogen atoms respectively. The arrow depicts the site of adsorption of Ce adatom.

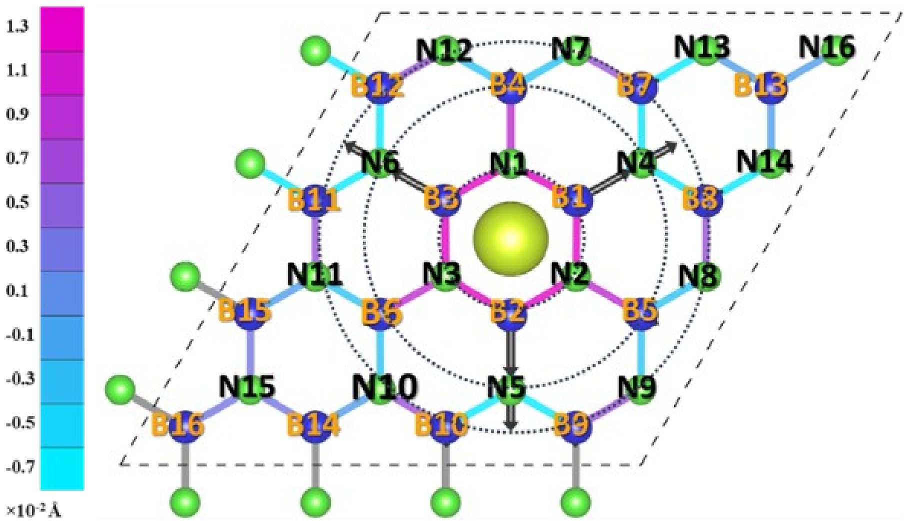

Fig. S4.2. Atomic displacement and bond stretching. Visual representation of xy-plane projected atomic displacement and bond elongation/shrinkages after Ce adsorption on h-BN. The thick black arrows are the displacement vectors scaled by a factor of 40. The dotted circles are a visual guide to mark the first, second and third shell of atoms closest to the HC site.

S5. Effect of Ce adsorption on charge distribution and interatomic distances.

Adsorption of Ce at the HC site brings about both structural and electronic changes in h-BN which are summarized in Figs. S5.1-5.3.

Fig S5.1. Bader charge analysis of Ce adsorbed on h-BN. Distribution of all atoms of Ce decorated h-BN according to electronic charge lost/gained. The red star symbols mark the excess charge on all B and N atoms in pristine h-BN without metal decoration.

Fig S5.2. Displacement of atoms from HC. xy-plane projected displacement of atoms from the central HC site.

Fig S5.3. Displacement of atoms from h-BN plane. The z-axis displacement of B, N atoms from the pristine h-BN basal plane after Ce adsorption.

A quantitative representation of the charge transfers as calculated by the Bader charge analysis is shown in Fig. S5.1. Overall, all B atoms continue losing charge to nearby N atoms. The sole Ce atom gives up about 0.58 electrons to the h-BN substrate. This excess charge is mostly taken up by the adjacent B and N atoms in the first neighboring shell (atoms labelled B1-3, N1-3 in Fig. S5.1). Compared to the charge uptake of B and N atoms in pristine h-BN (red star symbols in Fig. S5.1), charge transfer from Ce causes N atoms to become more negative and B atoms less positive. Adsorption of Ce over h-BN causes a few interesting structural changes in the substrate as well. Figures S5.2 and S5.3 depict the xy-plane projected and z-axis displacement of all B and N atoms.

Overall, the Ce adatom has excess positive charge and so do the nearest B atoms in first shell (B1-3 in Fig. S5.1). Contrarily, the nearest N atoms in the first shell (N1-3 in Fig. S5.1) have excess negative charge. Since Ce settles at the HC site, one expects the strain due to its addition to be uniformly distributed radially. However, the first shell N atoms do not shift as much as the first shell B atoms (N1-3 and B1-3 in Fig. S5.2). Therefore, one can rationally conclude, that the strain has two different origins: mechanical and electrostatic. Both mechanical strain and electronic repulsion between positively charged Ce and the adjacent B atoms add up and cause the first shell B atoms to be pushed out notably in xy-plane and slightly downwards (B1-3 in Fig S5.2 and S5.3). However, electronic attraction between oppositely charged Ce and N overcomes some of the structural strain and as a result, the nearest N atoms are only pushed downwards slightly (N1-3 in Fig. S5.3) with much less noticeable displacement in the xy-plane (N1-3 in Fig. S5.2). As a result of first shell B atoms' large displacement in xy-plane, the second shell N atoms witness a large push outwards (N4-6 in Fig. S5.2) as well as downwards (N4-6 in Fig. S5.3). The second shell B atoms (B4-6 in Fig. S5.3) attached to the first shell N atoms are pushed upwards. Thus, there is some warping of the h-BN substrate due to adsorption of a heavy element like Ce. Expectedly, B-N bonds closest to the HC site are elongated by ~0.8% (Fig. S4.2).

S6. Charge density difference on hydrogen adsorption.

Charge density difference isosurfaces are drawn for *n* hydrogen adsorbed Ce decorated h-BN.

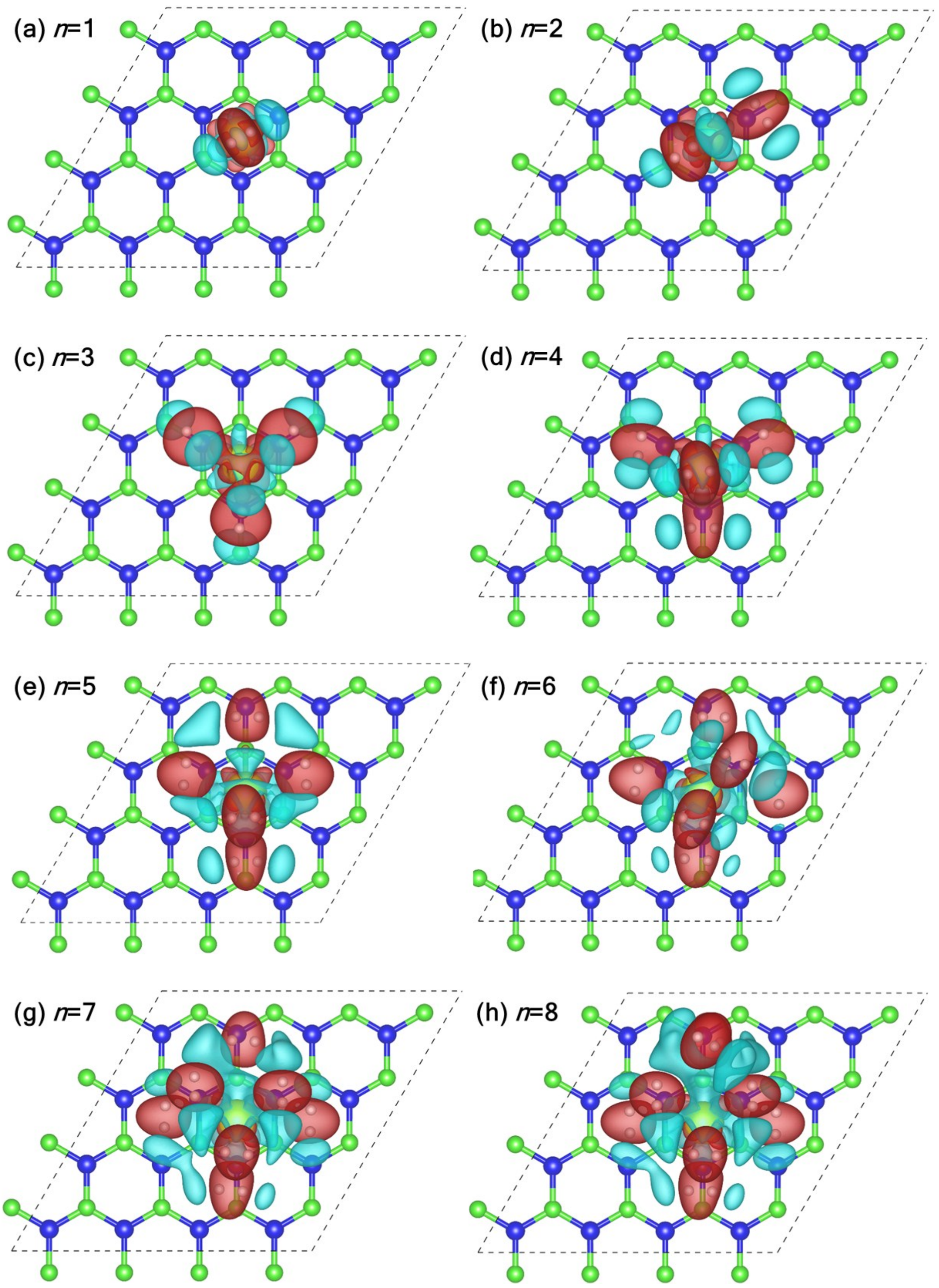

Fig S6.1. Charge density difference after hydrogen adsorption. Charge density difference distributions for *n*= 1-8 hydrogen molecules **(a-h)** adsorbed on Ce/h-BN. The isosurface level is set at 0.0045 electrons/Bohr^3. The red and cyan regions denote regions of charge accumulation and depletion respectively

References

[1] S. Lin, X. Ye, R. S. Johnson, and H. Guo, “First-principles investigations of metal (Cu, Ag, Au, Pt, Rh, Pd, Fe, Co, and Ir) doped hexagonal boron nitride nanosheets: stability and catalysis of CO oxidation,” *The Journal of Physical Chemistry C*, vol. 117, no. 33, pp. 17319–17326, 2013.

[2] C. Loschen, J. Carrasco, K. M. Neyman, and F. Illas, “First-principles LDA + U and GGA + U study of cerium oxides: Dependence on the effective U parameter,” *Physical Review B*, vol. 75, no. 3, Jan. 2007, doi: 10.1103/PhysRevB.75.035115.

[3] X. Liu *et al.*, “Adsorption and growth morphology of rare-earth metals on graphene studied by ab initio calculations and scanning tunneling microscopy,” *Physical Review B*, vol. 82, no. 24, p. 245408, 2010.

[4] B. Huang, R. Gillen, and J. Robertson, “Study of CeO2 and its native defects by density functional theory with repulsive potential,” *The Journal of Physical Chemistry C*, vol. 118, no. 42, pp. 24248–24256, 2014.

[5] M. Capdevila-Cortada, Z. Łodziana, and N. López, “Performance of DFT+ *U* Approaches in the Study of Catalytic Materials,” *ACS Catalysis*, vol. 6, no. 12, pp. 8370–8379, Dec. 2016, doi: 10.1021/acscatal.6b01907.

[6] I. Yeriskin and M. Nolan, “Doping of ceria surfaces with lanthanum: a DFT+ U study,” *Journal of Physics: Condensed Matter*, vol. 22, no. 13, p. 135004, 2010.

[7] S. Fabris, G. Vicario, G. Balducci, S. de Gironcoli, and S. Baroni, “Electronic and atomistic structures of clean and reduced ceria surfaces,” *The Journal of Physical Chemistry B*, vol. 109, no. 48, pp. 22860–22867, 2005.

[8] C. Ataca, E. Aktürk, S. Ciraci, and H. Ustunel, “High-capacity hydrogen storage by metallized graphene,” *Appl. Phys. Lett.*, vol. 93, no. 4, p. 043123, Jul. 2008, doi: 10.1063/1.2963976.

[9] C. Ataca, E. Aktürk, and S. Ciraci, “Hydrogen storage of calcium atoms adsorbed on graphene: First-principles plane wave calculations,” *Physical Review B*, vol. 79, no. 4, p. 041406, 2009.

[10] O. Faye, J. A. Szpunar, B. Szpunar, and A. C. Beye, “Hydrogen adsorption and storage on Palladium – functionalized graphene with NH-dopant: A first principles calculation,” *Applied Surface Science*, vol. 392, pp. 362–374, Jan. 2017, doi: 10.1016/j.apsusc.2016.09.032.

[11] Y. Liu, L. Ren, Y. He, and H.-P. Cheng, “Titanium-decorated graphene for high-capacity hydrogen storage studied by density functional simulations,” *Journal of Physics: Condensed Matter*, vol. 22, no. 44, p. 445301, Oct. 2010, doi: 10.1088/0953-8984/22/44/445301.

[12] Z. M. Ao and F. M. Peeters, “High-capacity hydrogen storage in Al-adsorbed graphene,” *Phys. Rev. B*, vol. 81, no. 20, p. 205406, May 2010, doi: 10.1103/PhysRevB.81.205406.

[13] D. P and S. Ramaprabhu, “Hydrogen storage in platinum decorated hydrogen exfoliated graphene sheets by spillover mechanism,” *Phys. Chem. Chem. Phys.*, vol. 16, no. 48, pp. 26725–26729, Nov. 2014, doi: 10.1039/C4CP04214J.

[14] E. Durgun, S. Ciraci, and T. Yildirim, “Functionalization of carbon-based nanostructures with light transition-metal atoms for hydrogen storage,” *Physical Review B*, vol. 77, no. 8, p. 085405, 2008.

[15] S. A. Shevlin and Z. X. Guo, “Transition-metal-doping-enhanced hydrogen storage in boron nitride systems,” *Applied Physics Letters*, vol. 89, no. 15, p. 153104, Oct. 2006, doi: 10.1063/1.2360232.

[16] M. Samolia and T. J. D. Kumar, “A conceptual DFT study of the hydrogen trapping efficiency in metal functionalized BN system,” *RSC Adv.*, vol. 4, no. 58, pp. 30758–30767, Jul. 2014, doi: 10.1039/C4RA03707C.
